# Efficacy and safety of self-expanding metallic stent placement followed by neoadjuvant chemotherapy and scheduled surgery for treatment of obstructing left-sided colonic cancer

**DOI:** 10.1186/s12885-020-6560-x

**Published:** 2020-01-28

**Authors:** Jia Gang Han, Zhen Jun Wang, Wei Gen Zeng, Yan Bin Wang, Guang Hui Wei, Zhi Wei Zhai, Bao Cheng Zhao, Bing Qiang Yi

**Affiliations:** 1 Department of General Surgery, Beijing Chaoyang Hospital, Capital Medical University, No. 8 South Gongti Road, Chaoyang District, Beijing, 100020 People’s Republic of China; 2 Department of Gastroenterology, Beijing Chaoyang Hospital, Capital Medical University, Beijing, China

**Keywords:** Left-sided colonic tumor, Intestinal obstruction, Self-expanding metallic stent, Neoadjuvant chemotherapy, Stoma

## Abstract

**Background:**

This study aimed to evaluate the safety and feasibility of self-expanding metallic stent (SEMS) followed by neoadjuvant chemotherapy prior to elective surgery for obstructing left-sided colon cancer.

**Methods:**

Eleven consecutive patients with obstructing left-sided colon cancer between May 2014 and November 2015 were included retrospectively. All patients received SEMS followed by neoadjuvant chemotherapy. The primary outcome measure was stoma and laparoscopic surgery.

**Results:**

Chemotherapy was with two cycles of CAPOX (54.5%) or three cycles mFOLFOX6 (45.5%). Median serum albumin and hemoglobin levels before surgery were significantly higher than before neoadjuvant chemotherapy (*p* = 0.01 and *p* = 0.008 respectively) and before SEMS (*p* = 0.01 and *p* = 0.003 respectively). Median bowel wall thickness proximal to the upper edge of tumor was significantly more before neoadjuvant chemotherapy than before stent (*p* = 0.003), and significantly less before surgery than before neoadjuvant chemotherapy (*p* = 0.003). No patient underwent stoma creation. Laparoscopic surgery was performed in nine (81.8%) patients. No local recurrence or metastases developed over median cancer-specific follow-up of 44 months (range, 37–55 months).

**Conclusion:**

SEMS followed by neoadjuvant chemotherapy prior to elective surgery appears to be safe and well tolerated in patients with obstructing left-sided colon cancer.

## Background

Approximately 10–30% of newly diagnosed colorectal cancer patients present with acute intestinal obstruction requiring urgent surgical treatment [1]. The risk of obstruction, which varies depending on the tumor site, is about 75% located in the left colon [2]. Postoperative mortality is much higher with emergency surgery than with elective surgery (15–30% vs. 1–5%), and the morbidity rate after emergency surgery (40–50%) is twice that of elective surgery [1, 3].

Although emergency surgery remains the main choice for patients with acute left-sided colon cancer obstruction, self-expanding metallic stent (SEMS) placement has been proposed as a bridge to surgery in patients with resectable disease [4, 5]. Several reviews have confirmed the feasibility and safety of SEMS for the treatment of colonic obstruction. The primary anastomosis rate is higher and the need for stoma creation is lower with SEMS plus elective surgery than with emergency surgery [6, 7]. Initial endoscopic SEMS decompression has been recommended by several bodies such as the American Society of Colon and Rectal Surgeons [8], National Comprehensive Cancer Network [9], World Society of Emergency Surgery [10], and Eastern Association for the Surgery of Trauma [11].

A retrospective study reported worse overall survival with SEMS and elective surgery than with emergency surgery in patients with left-sided malignant colon obstruction [5]. This has also been shown by a systematic review and meta-analysis [4]. Because of these reasons, the European Society of Gastrointestinal Endoscopy [12], Endoscopy and Cancer Committee of the French Society of Digestive Endoscopy, and the French Federation of Digestive Oncology [13] do not recommend the use of SEMS as a bridge to surgery. Thus, the ideal initial therapeutic approach for these patients remains controversial [1].

In practice, intestinal wall edema following stent placement increases the difficulty of surgery, and this could be a major problem if the interval between stent insertion and surgery is short (1–2 weeks) [14]. We hypothesized that prolonging the interval between stent insertion and surgery to 9–10 weeks, with neoadjuvant chemotherapy administered during this interval, would help improve outcomes. We reported the preliminary results in Chinese and case report previously [15, 16]. In this retrospective study we analyze the efficacy and safety of SEMS insertion followed by neoadjuvant chemotherapy and elective surgery in consecutive patients with obstructing left-sided colon cancer.

## Methods

We retrospectively reviewed the data of patients with acute obstructing left-sided colon cancer who underwent SEMS insertion followed by neoadjuvant chemotherapy prior to elective surgery at our center between May 2014 and November 2015. Clinically, obstruction was defined as complete failure to pass feces and gas and, radiologically, by distention of the colon in an abdominal computed tomographic (CT) scan. The eligibility criteria were 1) histologically proven adenocarcinoma located in the left colon (between the splenic flexure and 15 cm proximal to the anal margin) and 2) Eastern Cooperative Oncology Group (ECOG) performance status of from 0 to 2. Patients were excluded if they had 1) history of any other cancer, 2) multiple primary colorectal cancers, 3) distant metastases, 4) hereditary nonpolyposis colorectal cancer, or 5) familial adenomatous polyposis.

This study was approved by the institutional review board, and informed consent was obtained from all individual participants included in the study.

Primary tumor biopsy was performed during endoscopy for SEMS insertion. After biopsy, an uncovered SEMS (WallFlex; Boston Scientific Corporation, Natick, MA, USA) was inserted under fluoroscopic guidance as described previously [14]. Stent expansion and position was confirmed with abdominal radiography. Technical success was defined as successful deployment of the stent at the location of the stricture, and clinical success was defined as satisfactory bowel decompression within 24 h of stent insertion, with alleviation of clinical obstructive symptoms [17]. Colon wall thickness 10 cm proximal to the upper edge of tumor was measured on CT images at three time points: before stent insertion, before start of neoadjuvant chemotherapy, and before surgery.

Neoadjuvant chemotherapy was administered 1 week after successful stenting and decompression. Patients received either three cycles of mFOLFOX6 repeated every 2 weeks or two cycles of CAPOX repeated every 3 weeks. Toxicity was assessed according to the National Cancer Institute Common Toxicity Criteria (NCI-CTC, version 4.0).

Elective surgery was performed 2 weeks after completion of chemotherapy. Patients received laparoscopic or open surgery according to the surgeon’s decision and the patient’s condition. All tumor specimens were reviewed by two experienced pathologists. The tumor regression grade (TRG) was evaluated using the modified Dworak (mDworak) TRG system as follows: TRG 0 = no regression; TRG 1 = regression ≤25% of tumor mass (dominant tumor mass, with obvious fibrosis and/or vasculopathy); TRG 2 = regression > 25–50% of tumor mass (dominantly fibrotic changes, with a few, easily detected, clusters of tumor cells of groups); TRG 3 = regression > 50% of tumor mass (very few, difficult to detect, tumor cells in fibrotic tissue, with or without mucous substance); and TRG 4 = complete (total) regression (or response), with no detectable vital tumor cells [18, 19].

All patients received postoperative chemotherapy. Follow-up was arranged every 3 months for the first 2 years and then every 6 months for the next 3 years according to National Comprehensive Cancer Network (NCCN) guidelines.

### Statistical analysis

Patient demographics, disease characteristics, treatment details, SEMS-related complications, neoadjuvant chemotherapy–related adverse events, and postoperative complications were collected from the case records. Data were summarized as medians (and ranges). The Friedman test or Wilcoxon test were used for comparisons of the median values. Significance was defined as *p* <  0.05 or 0.05/k. Statistical analysis was performed using SPSS for Windows, version 22 (IBM Corp., Armonk, NY, USA).

## Results

Between May 2014 and November 2015, 14 patients with acute obstructing left-sided colon cancer underwent SEMS insertion in our department. Three patients were excluded from this analysis: two patients because of technical failure (inability to pass a guidewire) and one patient because of perforation (at 9 days after SEMS insertion, and just before neoadjuvant chemotherapy). These three patients received open surgery and diverting stoma. The remaining 11 patients comprised the study population; all underwent neoadjuvant chemotherapy after SEMS insertion. Table 1 shows the characteristics of the patients, tumors, and operations.

**Table 1:** Clinical characteristics of patients

Variables	*n* = 11
Age (y), median (range)	67 (43–72)
Male / female	7 (63.6)
BMI (kg/m^2^), median (range)	22.6 (20.1–26.6)
ASA score before stent placement, n (%)	
I or II	9 (81.8)
III	2 (18.2)
Tumor location, *n* (%)	
Sigmoid colon	7 (63.6)
Descending colon	2 (18.2)
Splenic flexure of colon	2 (18.2)
Smoking, *n* (%)	4 (36.4)
Alcohol consumption, *n* (%)	4 (36.4)
COPD, *n* (%)	1 (9.1)
Diabetes mellitus, n (%)	3 (14.3)
Hypertension, n (%)	5 (23.8)
Albumin (g/L), median (range)	33.3 (28.9–35.3)
Hemoglobin (g/L), median (range)	93.0 (70.0–144.0)
Prealbumin (g/L), median (range)	0.16 (0.12–0.22)
Bowel wall thickness at the beginning of the study (mm), median (range)	3.8 (3.0–7.0)
Preoperative chemotherapy regimen	
CAPOX	6 (54.5)
mFOLFOX6	5 (45.5)
ypT, *n* (%)	
T3	9 (81.8)
T4	2 (18.2)
ypN, *n* (%)	
N0	6 (54.5)
N1	4 (36.4)
N2	1 (9.1)
Tumor regression grade^a^	
1	4 (36.4)
2	5 (45.5)
3	2 (18.2)
Perineural invasion	0
Lymphvascular invasion	2 (18.2)
Tumor deposits	1 (9.1)
The time interval to elective operation (d), median (range)	69 (62–75)
Follow-up	44 (37–55)

*BMI* Body mass index, *ASA* American Society of Anesthesiologists, *COPD* chronic obstructive pulmonary disease, *CRM* circumferential resection margin

^a^Modified Dworak tumor regression grade system

**Fig. 1:**
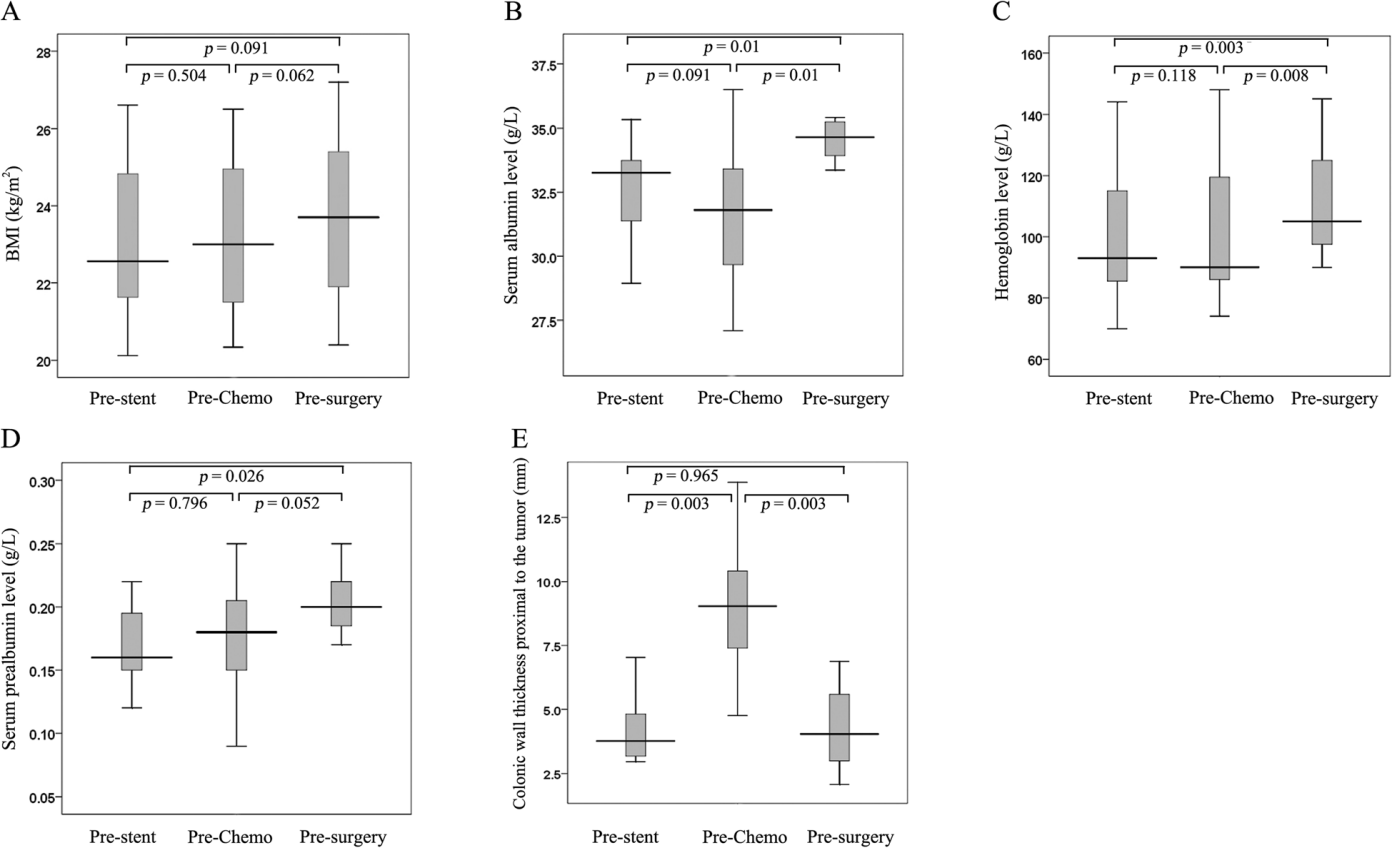
Comparison of characteristics of patients before SEMS, before neoadjuvant chemotherapy, and before surgery. **a**, There was no significant difference in BMI. **b**, Serum albumin before surgery was significantly higher than that before neoadjuvant chemotherapy (*p* = 0.01) and that before SEMS (*p* = 0.01). **c**, Hemoglobin level before surgery was significantly higher than that before neoadjuvant chemotherapy (*p* = 0.008) and that before SEMS (*p* = 0.003). **d**, There was no significant difference in prealbumin. **e**, Bowel wall thickness 10 cm proximal to the upper edge of tumor was significantly greater before neoadjuvant chemotherapy than before SEMS (*p* = 0.003), and significantly less before surgery than before neoadjuvant chemotherapy (*p* = 0.003). Statistical significance was at *p* < 0.017. *BMI* Body mass index, *Chemo* chemotherapy, *SEMS* self-expanding metallic stent

The general conditions of the patients before and after neoadjuvant chemotherapy were comparable. Median serum albumin and hemoglobin levels before surgery were significantly higher than the levels before neoadjuvant chemotherapy (34.7 g/L vs. 31.8 g/L, *p* = 0.01 to *p* <  0.017); and 105 g/L vs. 90 g/L, *p* = 0.008 to *p* < 0.017; respectively) and before SEMS (34.7 g/L vs. 33.3 g/L, *p* = 0.01 to *p* < 0.017; and 105 g/L vs. 93 g/L, *p* = 0.003 to *p* < 0.017; respectively). Median bowel wall thickness 10 cm proximal to the upper edge of tumor was significantly more before neoadjuvant chemotherapy than before SEMS (9.0 mm vs. 3.8 mm, *p* = 0.003 to *p* < 0.017), and significantly less before surgery than before neoadjuvant chemotherapy (4.0 mm vs. 9.0 mm, *p* = 0.003 to *p* < 0.017). (Table 2, Fig. 1).

**Table 2:** Comparison of patient status before and after neoadjuvant chemotherapy

Variables	Before stent	Before neoadjuvant chemotherapy	Before surgery	*p*
BMI (kg/m^2^), median (range)	22.6 (20.1–26.6)	23.0 (20.3–26.5)	23.7 (20.4–27.2)	0.178
Albumin (g/L), median (range)	33.3 (28.9–35.3)	31.8 (27.1–36.5)	34.7 (33.4–35.4)	0.003
Hemoglobin (g/L), median (range)	93.0 (70.0–144.0)	90.0 (74.0–148.0)	105.0 (90.0–145.0)	0.002
Prealbumin (g/L), median (range)	0.16 (0.12–0.22)	0.18 (0.09–0.25)	0.20 (0.17–0.28)	0.115
Bowel wall thickness (mm), median (range)	3.8 (3.0–7.0)	9.0 (4.8–13.9)	4.0 (2.1–6.9)	< 0.001

*BMI* Body mass index

Adverse events (mainly grade 1 and 2) occurred in five (45.5%) patients; grade 3 toxicity (diarrhea) was documented in only one (9.1%) patient. The most common toxicities were nausea (36.4%), anorexia (27.3%), leucopenia (18.2%), vomiting (18.2%), sensory neuropathy (18.2%), and skin hyperpigmentation (18.2%). No dose reduction was required during neoadjuvant chemotherapy (Table 3).

**Table 3:** Toxicities of neoadjuvant chemotherapy (n = 11)

Variables	Degree 1	Degree 2	Degree 3/4	
Leukopenia	2	0	0	
Neutropenia	1	0	0	
Thrombocytopenia	1	0	0	
Fever	0	0	0	
AST increased	1	0	0	
ALT increased	1	0	0	
Nausea	3	1	0	
Vomiting	2	0	0	
Diarrhea	1	0	1	
Anorexia	2	1	0	
Fatigue	1	0	0	
Sensory neuropathy	2	0	0	
Alopecia	0	0	0	
Mucositis oral	0	0	0	
Skin hyperpigmentation	2	0	0	

*ALT* alanine aminotransferase, *AST* aspartate aminotransferase

No patient had perineural invasion (PNI). Table 1 shows the pathological response (TRG grade or ypTNM stage) in surgical specimens. Among the 11 patients, two (18.2%) had TRG 3, five (45.5%) had TRG 2, and four (36.4%) had TRG 1. No patient had TRG 0 or TRG 4. While two (18.2%) patients were classified as good responders (TRG = 3), nine (81.8%) were classified as poor responders [18].

During the period of neoadjuvant chemotherapy, the sent migration and erosion rate was 0%. All patients underwent surgery after neoadjuvant chemotherapy. Table 4 lists the perioperative characteristics and the postoperative complications. No patient required stoma. Laparoscopic surgery was performed in nine (81.8%) patients and open surgery in two (18.2%) patients. One patient (the third patient in this series) required open surgery because of acute bowel obstruction precipitated by preoperative bowel preparation with polyethylene glycol. We checked the specimen during surgery and found there was no stent migration, while the sent was obstructed by the waste fiber. All subsequent patients received elemental diet for 3 days before surgery and 30 mL lactulose the day before surgery; no bowel obstructions occurred after this change in preoperative preparation. Open surgery was required for the other patient because of locally advanced tumor.

**Table 4:** Perioperative surgical variables and postoperative complications

Variables	*n* = 11
Surgery type, *n* (%)	
Laparoscopic surgery	9 (81.8)
Open surgery	2 (18.2)
Operative time (min), median (range)	160 (140–210)
Intraoperative blood loss, mL, median (range)	30 (20–100)
Stoma, n (%)	0
Time to first passage of flatus (h), median (range)	60 (36–88)
Postoperative defecation time (h), median (range)	64 (44–90)
Postoperative hospital stay, days, median (range)	7 (5–16)
Complications, *n* (%)	2 (18.2)
Wound infection, *n* (%)	1 (9.1)
Anastomotic leakage, *n* (%)	0
Ileus, *n* (%)	1 (9.1)
Anastomosis bleeding, *n* (%)	0
Intra-abdominal abscess, *n* (%)	0
Urinary system infection, *n* (%)	0
Pulmonary infection, *n* (%)	0
Deep venous thrombosis, *n* (%)	0
Reoperation, *n* (%)	0

No patient died in the postoperative period. Postoperative complications included wound infection (one patient; 9.1%) and ileus (one patient; 9.1%). Median cancer-specific follow-up was for 44 months (range, 37–55 months). No patient had local recurrence or metastases during follow-up.

## Discussion

This study aimed to determine the efficacy and safety of treatment of obstructing left-sided colon cancer with SEMS followed by neoadjuvant chemotherapy and elective surgery. The findings suggest that this approach is feasible and safe, and does not increase postoperative morbidity.

An important concern with neoadjuvant chemotherapy, especially when administered after SEMS for obstructive colorectal cancer, is the toxicity of the drugs used [20]. In our series, only one (9.1%) patient had a grade 3 toxicity (diarrhea). Neoadjuvant chemotherapy was generally well tolerated, and all patients were able to undergo surgery after completion of chemotherapy. Thus, two or three cycles of neoadjuvant chemotherapy after SEMS insertion appears to be safe.

A previous study reported a significant fall in serum albumin level despite sufficient nutritional intake when surgery was performed 2 weeks after stenting in patients with obstructive colorectal cancer; the authors suggested that SEMS insertion might increase the risk of anastomotic leakage [21]. Hosono *et al* [22] also reported decrease in serum albumin level in the interval between admission and surgery in 75% of patients treated with SEMS. In the present study, the serum albumin level decreased slightly after SEMS insertion, but then increased significantly in the interval between neoadjuvant chemotherapy and surgery. The hemoglobin level also increased significantly before surgery. Thus, it appears that the relatively longer interval before surgery allows improvement of the nutritional status of patients. This improvement probably contributes to the lower rate of postoperative complications.

The hypothesis driving the growing interest in the use of SEMS in colonic obstruction is that it can convert an emergency surgery into an elective one, and thus help minimize perioperative morbidity, aid restoration of bowel function, and decrease the need for a stoma [14, 21, 22]. However, the temporary stoma rate was much higher in patients treated with SEMS followed by elective surgery than in those treated directly with elective surgery (11% vs. 1%) [14]. The authors suggested that this may have been because the surgeons choose to make a stoma for preserving anastomotic integrity in patients with intraoperative intestinal wall edema as a result of previous colonic obstruction [14]. Preoperative evaluation of mucosal edema may help prevent anastomotic leakage [21]. We evaluated colonic wall thickness 10 cm proximal to the tumor and found that the thickness increased significantly 1 week after SEMS placement (before chemotherapy), and decreased significantly after chemotherapy. None of our patients required stoma creation, probably because of the improvement in physical condition and reduction of intestinal wall edema during the prolonged interval between SEMS and surgery.

Among patients receiving SEMS placement, 43.5–91% were successfully treated by laparoscopic surgery; this high success rate is partly attributable to intestinal decompression, which allows more efficient performance of laparoscopic surgery [21, 23]. As laparoscopic surgery is minimally invasive, short-term surgical outcomes are better [24]. In our study, laparoscopic surgery was successfully performed in nine (81.8%) patients. We suggest that neoadjuvant chemotherapy administered in the interval between SEMS placement and surgery does not decrease the possibility of success laparoscopic surgery; on the contrary, it might actually increase the chances of success.

Although neoadjuvant chemotherapy is not routinely administered in locally advanced colorectal cancer patients, several authors have demonstrated benefits with a neoadjuvant chemotherapy-alone approach [25, 26]. Studies have shown that neoadjuvant chemotherapy with CAPOX or FOLFOX is effective and safe in patients with locally advanced colon cancers [25, 26]. All patients in our study completed neoadjuvant chemotherapy without experiencing any major toxicities. Although no patient achieved complete pathologic response in our study, tumor reduction was achieved in all patients.

It was reported that tumor recurrence was more frequent in patients treated with SEMS [23, 27]. The poor outcomes may be related to the significantly increased levels of CK20 mRNA that have been detected in the peripheral circulation of colorectal cancer patients after endoscopic insertion of colonic stents; it is probably caused by tumor manipulation during guidewire insertion, dilatation, and stent deployment [28]. One of the disadvantages of colonic stenting is that it increases the risk of PNI [17, 21]. PNI is associated with decreased survival and is an independent predictor of poor outcome in colorectal cancer patients [29]. In our study, no patient had PNI. Moreover, no patient had local recurrence or metastases during follow-up. There were no tumor-related deaths during follow-up of 44 months. Therefore, we suggest that neoadjuvant chemotherapy after SEMS might lower the risk of PNI and thus help improve survival.

This study has some limitations. First, this was a single-center study with a small sample. Second, there was no comparison of patients treated with SEMS plus neoadjuvant chemotherapy and those treated with conventional SEMS. The comparison might reveal the real effect of new treatment on patients’ general conditions, operational variables and oncologic results compare with conventional SEMS treatment. Third, this was a retrospective case series, and a selection bias is therefore inevitable.

## Conclusions

This study suggests that SEMS followed by two or three cycles of neoadjuvant chemotherapy prior to elective surgery is a safe, effective, and well tolerated treatment approach for patients with left-sided obstructing colon cancer. The physical status of patients improves during the relatively longer interval between SEMS and surgery, thus probably contributing to reduction in postoperative complications. However, the follow up is too short to make meaningful conclusion about the oncologic safety of this approach. A prospective multicenter study on a large sample is needed to compare this treatment approach versus conventional SEMS and to determine the long-term oncological and patient outcomes.

## Data Availability

All the supporting data are available within the Supplementary Material.

## Abbreviations

CT: computed tomographic
ECOG: Eastern Cooperative Oncology Group
mDworak: modified Dworak
NCCN: National Comprehensive Cancer Network
NCI-CTC: National Cancer Institute Common Toxicity Criteria
PNI: perineural invasion
SEMS: self-expanding metallic stent
TRG: tumor regression grade
